# Streamlining the biodesulfurization process: development of an integrated continuous system prototype using *Gordonia alkanivorans* strain 1B†

**DOI:** 10.1039/d3ra07405f

**Published:** 2024-01-02

**Authors:** Tiago P. Silva, Susana M. Paixão, João Tavares, Filipe Paradela, Teresa Crujeira, José C. Roseiro, Luís Alves

**Affiliations:** a LNEG – Laboratório Nacional de Energia e Geologia, IP, Unidade de Bioenergia e Biorrefinarias Estrada do Paço do Lumiar, 22 1649-038 Portugal luis.alves@lneg.pt susana.alves@lneg.pt

## Abstract

Biodesulfurization is a biotechnological process that uses microorganisms as biocatalysts to actively remove sulfur from fuels. It has the potential to be cleaner and more efficient than the current industrial process, however several bottlenecks have prevented its implementation. Additionally, most works propose models based on direct cultivation on fuel, or batch production of biocatalysts followed by a processing step before application to batch biodesulfurization, which are difficult to replicate at a larger scale. Thus, there is a need for a model that can be adapted to a refining process, where fuel is being continuously produced to meet consumer needs. The main goal of this work was to develop the first bench-scale continuous biodesulfurization system that integrates biocatalyst production, biodesulfurization and fuel separation, into a single continuous process, taking advantage of the method for the continuous production of the biodesulfurization biocatalysts previously established. This system eliminates the need to process the biocatalysts and facilitates fuel separation, while mitigating some of the process bottlenecks. First, using the bacterium *Gordonia alkanivorans* strain 1B, continuous culture conditions were optimized to double biocatalyst production, and the produced biocatalysts were applied in batch biphasic biodesulfurization assays for a better understanding of the influence of different factors. Then, the novel integrated system was developed and evaluated using a model fuel (*n*-heptane + dibenzothiophene) in continuous biodesulfurization assays. With this system strain 1B surpassed its highest biodesulfurization rate, reaching 21 μmol h^−1^ g^−1^. Furthermore, by testing a recalcitrant model fuel, composed of *n*-heptane with dibenzothiophene and three alkylated derivatives (with 109 ppm of sulfur), 72% biodesulfurization was achieved by repeatedly passing the same fuel through the system, maintaining a constant response throughout sequential biodesulfurization cycles. Lastly, the system was also tested with real fuels (used tire/plastic pyrolysis oil; sweet and sour crude oils), revealing increased desulfurization activity. These results highlight the potential of the continuous biodesulfurization system to accelerate the transition from bench to commercial scale, contributing to the development of biodesulfurization biorefineries, centered on the valorization of sulfur-rich residues/biomasses for energy production.

## Abstract 2

Continuous BDS process prototype: integration of three steps – (1) biocatalyst production; (2) BDS; and (3) separation of desulfurized fuel/spent biocatalyst.

## Graphical abstract

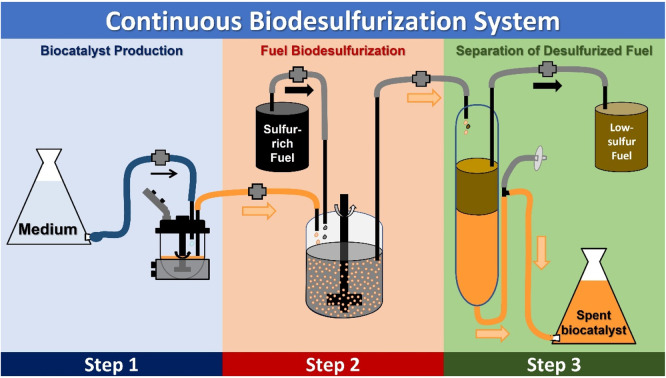

## 1. Introduction

Sulfur is one of the most important contaminants in fuels. It is abundant in oil and coal, and it can also be present in new-generation fuels if the biomass or residue used for their production also contains sulfur.^1^ Its concentration is strictly regulated, because when fuel is burnt it results in the release of sulfur oxides (SO_*x*_) and other sulfur molecules, leading to corrosion in motors and power generation systems, diverse health conditions and severe environmental problems (*e.g.*, acid rains).^2–4^ To prevent these issues, several desulfurization technologies have been studied over the years, but, when dealing with liquid fuels, the most common approach is hydrodesulfurization (HDS).^5^ For this process, molecular hydrogen (H_2_) is made to react with the sulfur atoms contained in the fuel, at high temperature and pressure, in the presence of metal catalysts, forming hydrogen sulfide (H_2_S) that can then be removed. While this method is very efficient at dealing with the simpler and linear sulfur molecules, it has difficulty reacting with sulfur contained in more complex organosulfur compounds such as dibenzothiophene (DBT), demanding greater energy spending, increasing the carbon footprint, and sometimes lowering the final quality of the fuel.^6^ These limitations have led researchers to look for alternatives or complementary processes, such as oxidative desulfurization, photocatalytic desulfurization, selective adsorption, extractive distillation or biodesulfurization (BDS).^7–11^ BDS is a biotechnological process dependent on the use of microorganisms, that act as biocatalysts, removing sulfur from fuels, and incorporating it into their biomass for their metabolic needs.^12^ It has the advantage of being used at low temperature (30 °C) and atmospheric pressure, without spending H_2_ or metal catalysts, while directly targeting the sulfur contained in the complex organosulfur molecules, without affecting fuel quality.^13^ Furthermore, in a biorefinery context, BDS can be coupled with the co-production of high added value products, such as biosurfactants, carotenoids, and other biomolecules, which can contribute to increase the sustainability of the bioprocess.^14–16^

Despite these interesting characteristics, BDS presents some limitations that are intrinsic to its biological nature: it has very low reaction rates, resulting in a slower process; it is inhibited by easily metabolized/easy access sulfur sources; the biocatalysts (the microorganisms) must be maintained under optimal conditions for their growth/metabolic activity and can be affected by the toxicity of compounds present in the fuels.^17–21^ Furthermore, there is currently a lack of a well-defined BDS model system, that defines how to produce and employ the biocatalyst, since different works often suggest opposite approaches to obtain optimal results. Additionally, most studies tend to focus on how to improve BDS rates, without considering the applicability of the proposed models. Amongst the different strategies suggested, some propose the utilization of immobilized biocatalysts, which are directly applied to the fuel.^22^ This increases resistance to toxicity and facilitates the separation of the fuel in the downstream process. However, immobilization makes the process more complex and often limits access to the sulfur compounds present in the fuel and the oxygen needed for the BDS pathway, while also resulting in a local increase of BDS products, consequently leading to lower BDS activities.^23^ Therefore, most authors agree that BDS will require the use of biocatalysts in the form of free cells, applied to a biphasic system, in which there will be a mixture between an aqueous phase (*i.e.*, culture medium containing the biocatalyst) and an organic phase (the sulfur-rich fuel). This presents problems of its own, such as low mass transfer between phases, toxicity and the formation of strong emulsions between cells, water and fuel, which have been at the center of many studies.^24–26^ This last point is especially critical, since it hinders continuous operation of the BDS process, leading to increased downstream processing costs.

Within this approach there are multiple variations. Some authors suggest direct cultivation of biocatalyst in the fuels, supplementing the aqueous phase with the nutrients needed to promote microbial proliferation.^27^ However, this can lead to lower growth and BDS rates, despite having shown success in sulfur removal. Furthermore, this strategy is avoided by many authors due to the risk of fuel contamination with unwanted microorganisms, which could lead to fuel degradation caused by hydrocarbon consumption.^28^

Currently, many of the most studied approaches to the BDS process separate biocatalyst production and BDS into two independent steps.^21,29^ This allows the production of high amounts of biocatalyst with BDS activity, followed by application in the form of resting cells, which actively remove sulfur even without proliferation. This method accelerates biocatalyst production, increases BDS rates and limits the risk of contamination with other microorganisms, while also allowing the optimization of different conditions and designs for each stage.

However, despite the plethora of designs and strategies, the BDS always presented one limitation: the absence of a defined method for the continuous production of BDS biocatalysts using simple sulfur sources. As such, in most works, both biocatalyst production and BDS were optimized to operate in batch conditions, often separated by complex processing steps. This resulted in the development of systems and optimization strategies which are time-consuming and more difficult to scale-up.

Recently, an innovative method was reported for the continuous cultivation of biocatalysts with BDS activity using easy-access and low-cost sulfur sources.^20^ This method allowed the production of biocatalysts for BDS using inhibitory sulfur sources, without the need for induction, specific carbon sources, nor genetic manipulation, and without reducing BDS activity nor biomass production. The development of this continuous production method opened the possibility of integrating biocatalyst production and BDS into a single continuous process, which can be used to streamline the BDS process, bypassing many of its limitations, making it closer to large scale application.

*Gordonia alkanivorans* strain 1B is a bacterium that has been extensively studied for its ability to perform BDS. It can remove sulfur not only from DBT, without affecting its carbon content, but it can also act on other organosulfur molecules, such as benzothiophene and even DBT derivatives, which are especially recalcitrant to desulfurization.^30–32^ Despite being a fructophilic bacterium, which prefers fructose to glucose as a carbon source, it has shown substantial metabolic plasticity, being able to grow on different sugars and alcohols and even complex carbon sources based of agro-industrial residues, such as recycled paper sludge, molasses, or different used cooking oils.^14,32–35^ It can also produce high added value products, such as carotenoids (*i.e.*, astaxanthin, canthaxanthin and lutein) and biosurfactants/bioemulsifiers with properties comparable to some commercial detergents.^14–16,36^ These characteristics make strain 1B especially interesting for modern biotechnological applications, allowing the exploration of different feedstocks for cultivation and the coproduction of multiple products, potentially increasing the viability of a future BDS biorefinery. Moreover, in Silva *et al.*,^20^ when the strain 1B was cultivated using the novel continuous production method, it demonstrated greater biomass production and biodesulfurization rates than *Rhodococcus erythropolis* D1 following the same cultivation method.

However, despite the promising results, the study of Silva *et al.*^20^ was mostly centered on continuous biocatalyst production and BDS was always preceded by a biocatalyst washing and concentration step, which made it impossible to perform a direct integration of this production method with a continuous BDS process. Furthermore, BDS was only assessed in batch conditions through resting cells assays in which DBT was in aqueous suspensions. These conditions are substantially different from real fuel BDS; therefore, it is important to understand how the strain 1B behaves in biphasic assays, accounting for both BDS and subsequent separation of biocatalyst from fuel.

In this context, this work aimed to take advantage of the continuous biocatalyst production and develop a bench-scale continuous BDS system prototype, to streamline the BDS process using cells of *G. alkanivorans* strain 1B as biocatalysts. The first step consisted of adapting continuous cultivation conditions to increase biocatalyst production and eliminate the processing steps (washing and concentration), while limiting the spending/waste of nutrients. Then, cells resulting from the optimal continuous biocatalyst production system were used in two experimental designs (EDs) to study how strain 1B would react in batch biphasic resting cells assays. Based on the data set obtained, a novel BDS system was developed combining continuous biocatalyst production, continuous biphasic desulfurization, and continuous separation of desulfurized fuel. This system was studied using different model fuels, with initial optimization of key parameters (working volume, DBT concentration and fuel : water ratio (*i.e.*, organic : aqueous ratio)). After optimization, the continuous BDS system proposed herein, was evaluated towards its potential to desulfurize different fuels (recalcitrant model fuel; real fuels: pyrolysis/crude oils).

## 2. Materials and methods

### 2.1. Reagents and fuels

DBT (99%) was from Acros Organics, 4-methyl DBT (4-mDBT) (96%), 4,6-dimethyl DBT (4,6-dmDBT) (97%) and 4,6-diethyl DBT (4,6-deDBT) (97%) were from Aldrich Chem. Co. 2-Hydroxybiphenyl (2-HBP) was from Sigma, dimethylformamide (DMF) was from Riedel-de-Haën and *n*-heptane was from Carlo Erba (99%). Real fuels consisted of crude oils and a pyrolysis oil. Samples of crude oils (sour = Iranian light sour oil, with 16 000 ppm of sulfur (S); and sweet = Mondo sweet oil, with 4080 ppm of S) were provided by Galp Energia, and the pyrolysis oil (Pyr) was produced in our laboratory (LNEG), from a blend of scrap tire/plastic (polypropylene) residues [30/70, % (m m^−1^)], which contained 1190 ppm of S.

### 2.2. Microorganism

This study was conducted using the bacterium *Gordonia alkanivorans* strain 1B, which was maintained in a sulfur-free mineral (SFM) culture medium, as described by Alves and Paixão,^32^ supplemented with 5 g L^−1^ of fructose as the carbon source (C-source) and 150 μM of DBT as sulfur source (S-source). Prior to bioreactor inoculation, *G. alkanivorans* strain 1B was transferred to a 500 mL shake-flask with 100 mL SFM and left to grow for 72 h at 150 rpm 30 °C (inoculum).

### 2.3. Biocatalyst production

Biocatalysts (bacterial cells) were produced in a chemostat, adapting the method described in Silva *et al.*^20^ A chemostat of 3.3 L (News Brunswick Inc, Bioflo III, USA) was used with a 0.7 L working volume. Dilution rate was kept at 0.0675 ± 0.0015 h^−1^, and aeration and agitation rates were maintained at 2 vvm (volume of air per volume of liquid per minute) and 425 rpm (revolutions per minute), respectively. Bioreactor was inoculated with 50 mL of inoculum, under aseptic conditions, and left to grow under batch conditions for 72 h, before starting the continuous culture. To increase biomass production, culture medium was also adjusted to the following formulation for 1 L: 2.2 g NH_4_Cl, 1 g KH_2_PO_4_, 1 g Na_2_HPO_4_·2H_2_O, 0.0852 g MgCl_2_·6H_2_O, 0.063 g Na_2_SO_4_, 0.25 mL micronutrient solution^32^ and 20 g fructose. Continuous biomass production was kept at 8.25 ± 0.75 g L^−1^ (dry cell weight – DCW) over several months.

### 2.4. Experimental designs (EDs)

To study how the BDS activity of strain 1B cells was influenced by the fuel : water ratio, *i.e.*, the sulfur concentration in the fuel (organic phase) and the biocatalyst concentration in the water (aqueous phase), two different EDs were performed following a Doehlert uniform shell design for two factors,^37^ considering each test mixture of *n*-heptane with DBT as a model fuel. For each ED, seven conditions were selected and tested in duplicate.

In the first experimental design (ED1), the two factors tested were: *X*_1_ – the ratio of *n*-heptane : water, between 1 : 9 (0.1) and 9 : 1 (0.9), and *X*_2_ – initial DBT concentration in the *n*-heptane phase, between 0.250 mM and 2 mM. For ED1, the aqueous phase contained the biocatalyst suspension at a concentration of 8.4 g L^−1^ (DCW). In the second experimental design (ED2) the two factors tested were: *X*_1_ – the ratio of *n*-heptane : water, between 0.1 and 0.9, and *X*_2_ – cell (biocatalyst) concentration in the water phase, between 5 g L^−1^ and 20 g L^−1^ (DCW). In ED2, DBT concentration in the *n*-heptane phase was kept constant at 1.13 mM. For both EDs, response was evaluated in terms of 2-HBP produced (the result of DBT biodesulfurization), in μM, and samples were collected at 3 and 6 h. Results were analyzed as previously described by Fernandes *et al.*,^38^ and a second-order polynomial model (eqn (1)) was used to express the response:

1 *Y*_*i*_ = *β*_0_ + *β*_1_*X*_1_ + *β*_2_*X*_2_ + *β*_12_*X*_12_ + *β*_11_*X*_12_ + *β*_22_*X*_22_

where *Y*_*i*_ is the response from experiment *i*, *β* are parameters of the polynomial model and *X* is the experimental factor level (coded units). The response factors obtained were then used to draw response surfaces using the SigmaPlot software (version 14).

#### 2.4.1. Biocatalyst preparation

Cells produced in the chemostat under optimal conditions were collected to ice and centrifuged at 6000×*g*, 4 °C for 20 min to obtain a concentrated cell suspension. The cell-free supernatant obtained in the centrifugation was then used to dilute the cell suspension to reach the different cell concentrations determined by the ED.

#### 2.4.2. BDS assay

According to the conditions established by the respective ED, the *n*-heptane containing DBT (model fuel) and the bacterial biomass suspension (biocatalysts in the aqueous phase), totaling 4 mL of combined volume, were transferred to 30 mL screw-cap glass tubes, ensuring enough space for mixture and aeration. The tubes were then placed horizontally in an orbital incubator at 30 °C and 150 rpm. At 3 h and 6 h, two tubes of each condition were collected and placed in ice to stop the reaction. These sampling times were selected based on the previous work, which demonstrated that BDS rates can be over-estimated in the first 30 to 60 min, but are typically stable during the subsequent 4 to 5 h, to highlight differences in BDS speed. Afterwards, the tubes were left resting vertically for 1 h at 4 °C, to allow phase separation. Each tube was treated as an individual sample, to avoid influencing the liquid ratios. The upper layer was collected and centrifuged at 15 000×*g* for 5 min, and the cell-free *n*-heptane layer was analyzed through HPLC for 2-HBP quantification.

### 2.5. Continuous BDS system

**Fig. 1:**
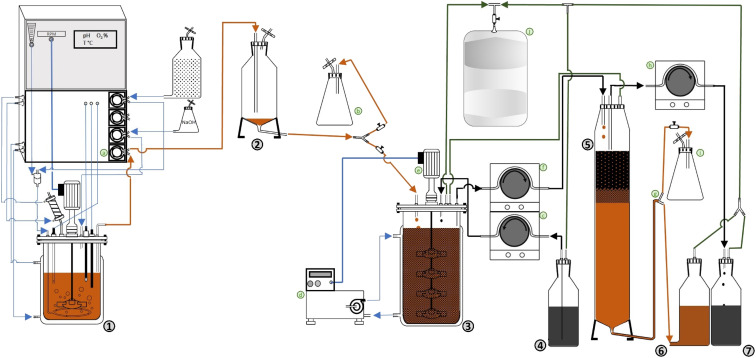
Schematic representation of the continuous BDS system prototype (not to scale). (1) – Continuous biocatalyst production in a chemostat; (2) – degasser; (3) – biodesulfurization vessel; (4) – fuel container; (5) – separation column; (6) – used biocatalyst (cell) container; (7) – biodesulfurized fuel container; (a) – peristaltic pump, connecting (1) to (2); (b) – collection point; (c) – peristaltic pump connecting (4) to (3); (d) – thermostatic bath; (e) − propellers; (f) – peristaltic pump connecting (3) to (5); (g) – leveling system; (h) – peristaltic pump connecting (5) to (7); (i) – pressure relief flask; (j) – gas-tight bag.

A bench-scale continuous BDS system, presented in Fig. 1 and S1 (ESI),† was developed based on the continuous production of biocatalysts with BDS activity. The system is composed by three separate main steps: (i) biocatalyst production, (ii) biodesulfurization, (iii) separation. Fig. 1 is a schematic of the prototype, where numbers represent the main components of the system, and the letters represent secondary components needed to transfer cells/fuel from one step to another or regulate conditions. Fig. S1† is a photo of the actual prototype used in the lab for the experiments described.

(i) Biocatalyst production

The first step consists of the continuous production of cells with BDS activity at a fixed rate and constant cell concentration (in the present case 0.049 ± 0.001 L h^−1^ and 8.25 ± 0.75 g L^−1^ DCW, respectively). Regarding Fig. 1, cells produced in a chemostat (1) are continuously collected *via* peristaltic pump (a), pass through a degasser vessel (2), for separation of cells and air, and are then transferred to the biodesulfurization vessel (3). The present system also possesses a collection point (b), for sampling and system manipulation before BDS.

(ii) Biodesulfurization

The second step consists of the mixing of biocatalysts and fuel at a fixed ratio, under optimal conditions for BDS (Fig. 1). So, fuel is transferred from the container (4) to the biodesulfurization vessel (3) (in this case, with 0.5 L maximum volume), using a peristaltic pump (c). At the biodesulfurization vessel (3) both cells and fuel continuously drip at a fixed ratio and are maintained at optimum temperature and agitation to ensure maximum BDS (in this work, 30 °C and 300 rpm, respectively). Temperature and agitation are ensured using a thermostatic bath (d) and a propeller (e), respectively. Volume is maintained constant *via* a leveling system, composed of a stainless-steel collection tube connected through a flexible tube to a peristaltic pump (f), which continuously collects the fuel + water + cells mixture to the separation column (5). In this BDS system, volume/reaction time can be adjusted by changing the height of the collection tube and the fuel : water ratio can be adjusted by changing the fuel input rate and/or the biocatalyst input rate. The headspaces of the biodesulfurization vessel and separation column are connected *via* tubing, to avoid injecting air into the separation column. Sampling can be performed *via* a sampling port in the biodesulfurization vessel.

(iii) Separation

The third and final step of the BDS system (Fig. 1) is the continuous separation of fuel from water + cells. The mixture of fuel + water + cells is continuously dripped from above into the separation column (5), with a volume adjusted to allow phase separation (in this case 0.450 L maximum volume).

The biodesulfurized fuel, which forms the upper phase, is then collected *via* a surface level tube in the upper part of the column, using a peristaltic pump (h) into a container (7). The lower layer, containing water and cells, is drained through the lower end to a container vessel (6). A leveling system (g) is used to adjust column volume and maintain the relative height of both phases to guarantee proper separation. The leveling system (g) is composed of a “T” connector with one end receiving the aqueous phase from separation column (5), one end connecting to the cell container vessel (6), and the last end connecting to a pressure relief flask (i). In this system the separation column (5) is heated with the same thermostatic bath (d) used for the biodesulfurization vessel (3), to decrease viscosity and destabilize emulsions, facilitating phase separation.

Any emulsion carried to the cell container (6) can be easily separated once the container is full. Alternatively, the system can be adjusted so that phase emulsion is collected in the oil container, by adjusting the height of the leveling system (g). In both cases, the emulsion can then be transferred to another separation column to repeat the separation process, or it can be retrieved and subjected to a soft centrifugation at 424×*g* for 10 min.

To facilitate system handling and avoid losses due to evaporation, the entire system was made airtight. To collect any volatiles and prevent either vacuum or excess pressure in different points of the process, resulting from action of the simultaneous pumps, the multiple headspaces (from the second and third steps) were connected to a single gas-tight bag (j). The flexible tubes used to transfer the fuels (*e.g.*: in (c), (f) and (h)), as well as those connecting to the bag (j) were made of viton, to ensure reduced interaction with the fuels, or loss *via* evaporation.

#### 2.5.1. Optimization of operating conditions

Considering as base conditions a volume of 0.215 ± 0.013 L and 34.5% *n*-heptane with 0.5 mM of DBT, three separate factors were selected to optimize the operating conditions of the BDS system described above: working volume (20–405 mL)/retention time (16.2 min–5.5 h), ratio of organic : aqueous phases (1 : 9–7.5 : 2.5), and DBT concentration (0.125 mM–2 mM). For all cases *n*-heptane with DBT was selected as a model fuel, and the biocatalysts (cells of *G. alkanivorans* strain 1B) were continuously produced under similar conditions. Biocatalyst flow rate (and concentration), temperature and agitation were kept constant, as described above, and pH was not controlled. Between assays, the biodesulfurization vessel, separation column, cells and fuel containers and tubes were cleaned, to ensure there was no influence of previous tests. At the start of each assay the biodesulfurization vessel was emptied, so that cells and fuel drip at the correct ratio from the start. Samples were taken directly from the sampling port in the biodesulfurization vessel, after at least six turnovers of the working volume to ensure a stable response. Optimized conditions were then used in all subsequent BDS assays.

#### 2.5.2. Fuel preparation

The different real fuels (sour, sweet and Pyr) tested in the BDS system, under optimum conditions, were first diluted in *n*-heptane to increase the sample volume and adjust the initial sulfur concentration to approximately 100 ppm. After dilution, fuels were subjected to centrifugation, 30 min at 6800×*g*, 25 °C, to remove particulates and avoid a false overestimation of BDS.

#### 2.5.3. Sequential BDS

To demonstrate the potential of the continuous system, an assay was developed in which a model fuel was subjected to several rounds of continuous BDS, simulating a sequential process. With this goal, another model fuel was prepared, mimicking an extremely recalcitrant fuel, by supplementing *n*-heptane with DBT, 4-mDBT, 4,6-dmDBT and 4,6-deDBT, each in equal parts, to obtain ≈109 ppm of total sulfur. This fuel was repeatedly fed to the novel BDS system for a total of 13 repetitions. After each cycle of BDS, at least 20 mL of sample were collected, for different analysis, and kept at −20 °C in screwcap tubes, to prevent evaporation.

### 2.6. Analytical methods

#### 2.6.1. Culture monitorization

Biocatalyst production was monitored, as described in Silva *et al.*,^20^ through optical density (OD_600_) and dry cell weight (DCW). Sugar consumption was evaluated through HPLC, pH and dissolved oxygen through electrodes in the chemostat vessel and CO_2_ produced and O_2_ consumed through a gas analyzer.

#### 2.6.2. HPLC quantification of BDS

An Agilent 1100 series HPLC system (Agilent Technologies, Germany) equipped with a diode array detector (DAD), reading at 220 nm was used to determine the concentration of DBT, 2-HBP and their derivatives in the different model fuels. Calibration curves were regularly performed using standards for 2-HBP, DBT, 4-mDBT, 4,6-dmDBT and 4,6-deDBT dissolved in *n*-heptane, with a minimum concentration of 5–7.5 μM, and the minimum quantification limit was determined to be approximately 1 μM. The remaining desulfurized DBT derivatives were evaluated in terms of absolute area, as no standard was available. Samples were run through an Agilent Eclipse PAH (5 μm 4.6 × 250 mm) column at 25 °C with water and acetonitrile (ACN) at 0.420 mL min^−1^ as mobile phase. For samples containing only DBT and 2-HBP dissolved in *n*-heptane the flow was kept for 0.9 min at 60% water 40% ACN. From 0.9 to 12 min water was gradually reduced to 0% with a respective increase in ACN to 100%, these conditions were maintained from 12 to 14.50 min. From 14.50 to 22 min, water was gradually increased to 60% with the respective decrease in ACN, and from 22 to 23 min conditions were maintained, reaching the end of the run. For samples containing a mixture of several DBTs the flow was kept for 0.9 min at 60% water 40% ACN. From 0.9 to 24 min water was gradually reduced to 0% with a respective increase in ACN to 100%, these conditions were maintained from 24 to 29 min. From 29 to 44 min water was gradually increased to 60% with the respective decrease in ACN and from 44 to 46 min conditions were maintained, reaching the end of the run. Prior to analysis, all samples were centrifuged at 15 000×*g* for 10 min and placed in closed vials with solvent resistant septa.

#### 2.6.3. Total sulfur determination

Total sulfur concentration was determined by X-ray fluorescence (XRF) using an AXIOS sequential wavelength dispersive spectrometer, fitted with a 4 kW generator and a rhodium anode X-ray super sharp tube and controlled by PANalytical SUPER Q software, at the Laboratory of Biofuels and Biomass (LBB), an accredited laboratory, according to NP EN ISO/IEC 17025: 2018, at LNEG (Lisbon, Portugal).

Based on the accredited standard ISO 20884, an extended methodology was developed and validated, including sulfur measurements in pyrolysis oils and crude oils in *n*-heptane matrix, as is the case of this work. The values of sulfur presented are the mean values obtained from duplicate analysis (±standard deviation) and the minimum quantification limit was approximately 11.0 mg kg^−1^. At least two samples of each biodesulfurized fuel and an initial sample of the diluted fuel were collected. Samples were centrifuged at 15 000×*g* for 10 min, at 25 °C, to remove solid impurities that influence the X-ray technique. As previously stated, samples were collected after six turnovers of the culture medium, and with at least 1 turnover between them.

#### 2.6.4. GC-MS

An Agilent 8890 GC was used to detect and identify the compounds resulting from the biodesulfurization of the different DBT derivatives. It was, equipped with a 5977B GC/MSD and an Agilent DB-5ms 30 m × 250 μm × 0.25 μm column. Column flow was 1 mL min^−1^, with helium as carrier gas, injection split 10 : 1, pressure 8.2 psi and inlet temperature 250 °C. Oven temperature started at 60 °C, with a heating ramp of 15 °C min^−1^ up to 100 °C, 25 °C min^−1^ up to 260 °C, with a hold time of 15 min, and 25 °C min^−1^ up to 300 °C with a hold time of 5 min. MS transfer line temperature was 250 °C, MS source temperature was 230 °C and MS quad temperature was 150 °C. MS scan time segments were, from 0 to 7 min: masses read from 10 to 250, and from 7 min to end of run: masses read 40 to 340. The scan speed was constant at 781 u s^−1^.

## 3. Results and discussion

### 3.1. Continuous culture conditions

To directly integrate continuous biocatalyst production and BDS stages, it was necessary to increase biocatalyst concentration (*i.e.*, cells biomass), while limiting nutrient wasting without lowering BDS rates. Hence, some modifications were implemented to the culture conditions proposed by Silva *et al.*^20^ for the continuous production of biocatalysts with BDS activity:

(1) Carbon and sulfur source concentrations were doubled, maintaining the carbon/sulfur ratio previously described by Silva *et al.*,^20^ which demonstrated that at 10 g per L of fructose and 22 mg per L SO_4_^2−^, both carbon and sulfur limited biomass production. Therefore, to increase biomass production without compromising BDS, it was necessary to increase these components while maintaining this ratio and guaranteeing that there was no accumulation of sulfur, to avoid inhibition.

(2) The relative concentrations of NH_4_Cl, MgCl_2_·6H_2_O and micronutrient solution were adjusted. Indeed, the culture medium employed by Silva *et al.*^20^ was a direct adaptation of SFM medium,^32^ which was developed to ensure excessive amounts of these components to prevent nutrient limitation during shake-flask assays. However, Pacheco *et al.*^39^ demonstrated this excess was not only an unnecessary added cost, but it also negatively affected growth and BDS rates; and, as such, the relative concentration of these nutrients was reduced in accordance with those findings.

(3) The relative concentrations of KH_2_PO_4_ and Na_2_HPO_4_·2H_2_O were also adjusted. As mentioned above, the original SFM medium was developed to be used in shake-flask assays, as such the main role of these phosphate compounds was to act as a buffer and reduce pH fluctuations. Considering that in the chemostat pH was maintained by addition of NaOH on demand and the constant dilution with fresh medium, the buffer components were reduced, to avoid nutrient wasting.

So, all the mineral salts of the culture medium were adjusted in proportion considering the duplication of C- and S-sources. The final culture medium formulation is presented in Section 2.3.

(4) Dilution rate was adjusted to 0.0675 ± 0.0015 h^−1^, to allow complete sulfate consumption and avoid inhibition of the BDS activity of the produced biocatalysts.

With these adjustments it was possible to increase biomass concentration, from 4.5 ± 0.5 g L^−1^ to 8.25 ± 0.75 g L^−1^ (DCW), while maintaining BDS activity at a similar rate. This allowed the continuous production of biomass, suspended in a sulfur-free culture broth, which is ready for direct application in the BDS process, without the need for further processing, such as concentration, or washing with buffer solution. Indeed, this approach resulted in the production of a continuous stream of biocatalysts that can directly act as resting cells, maintaining their BDS activity.

### 3.2. Experimental designs (EDs)

Due to the reduced knowledge on the behavior of strain 1B under biphasic conditions, before designing a BDS system prototype it was necessary to better understand how different factors may influence desulfurization in a biphasic setting. Taking into consideration that this system would integrate the continuous biocatalyst production method described above, three factors were selected for these initial studies: (1) organic : aqueous ratio – higher ratios are ideal for industrial purposes, however, they can have adverse effects on biological activity and mass transfer, which could affect BDS activity;^40^ (2) DBT concentration – the response to different concentrations can determine the potential of the biocatalyst, as DBT and especially the 2-HBP, resulting from its desulfurization, are known to be toxic at higher levels.^26,41^ In Silva *et al.*,^20^ BDS by strain 1B was studied with 250 μM of DBT, as such it was important to understand its behavior/potential to deal with greater concentrations; and (3) biocatalyst concentration – effectiveness at low concentrations would be ideal, but the increase in concentration could also mitigate adverse effects caused by toxicity and/or compensate lower biocatalyst activity.^26^

In this context, two EDs were performed using *n*-heptane with dissolved DBT as a sulfur-rich model fuel (organic phase) and cells suspended in water as biocatalysts (aqueous phase). The assays were carried out in closed screwcap tubes to assess how the organic : aqueous phases ratio (designated as *n*-heptane : water ratio), influenced DBT BDS when conjugated with different DBT concentrations (ED1) and different biocatalyst concentrations (ED2).

**Fig. 2:**
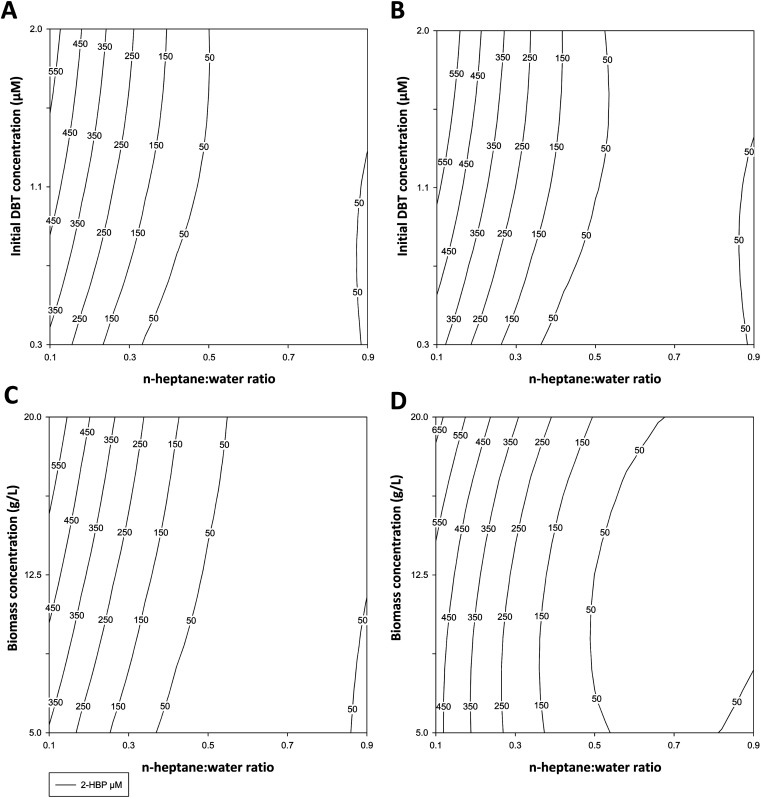
Response surfaces for 2-HBP production (μM) based on the responses obtained in ED1 at 3 h (A) and 6 h (B) [for the factors *X*_1_ – the ratio of *n*-heptane : water, between 1 : 9 (0.1) and 9 : 1 (0.9), and *X*_2_ – initial DBT concentration in the organic phase, between 0.250 mM and 2 mM], and ED2 at 3 h (C) and 6 h (D) for the factors [*X*_1_-the ratio of *n*-heptane : water, between 0.1 and 0.9, and *X*_2_-cell (biocatalyst) concentration in the water phase, between 5 g L^−1^ and 20 g L^−1^ (DCW)].

Table 1 indicates the conditions tested and the experimental results obtained (2-HBP produced, in μM) for ED1 and ED2. These results were applied to the polynomial model to obtain the response surfaces and the response factors presented in Fig. 2A–D and in Table 2, respectively.

**Table 1:** Doehlert distribution for two factors: ED1 – *X*_1_: ratio of *n*-heptane (with the DBT) : water (cell suspension with 8.4 g L^−1^), between 1 : 9 (0.1) and 9 : 1 (0.9), and *X*_2_: initial DBT concentration in the organic phase, between 0.25 mM and 2 mM, and ED2 – *X*_1_: ratio of *n*-heptane (with 1.13 mM DBT):water (cell suspension), between 1 : 9 (0.1) and 9 : 1 (0.9), and *X*_2_: cell (biocatalyst) concentration in the aqueous phase, between 5 g L^−1^ and 20 g L^−1^ (DCW). Responses were evaluated in terms of 2-HBP concentration in the organic phase (μM), after 3 h and 6 h. For each ED seven conditions were tested in duplicate (14 tests), the results presented are an average of the duplicates

ED (#)	Factors			Response	
	Ratio *n*-heptane : water	DBT (mM)	Biocatalyst (g L^−1^ DCW)	2-HBP (μM)	
				3 h	6 h
ED1	1 : 1 (0.5)	1.13	8.4	31	54
	9 : 1 (0.9)	1.13		3	7
	1 : 9 (0.1)	1.13		550	625
	7 : 3 (0.7)	1.88		24	31
	3 : 7 (0.3)	0.37		42	71
	7 : 3 (0.7)	0.37		9	17
	3 : 7 (0.3)	1.88		202	240
ED2	1 : 1 (0.5)	1.13	12.5	40	49
	9 : 1 (0.9)		12.5	6	10
	1 : 9 (0.1)		12.5	534	532
	7 : 3 (0.7)		19	21	34
	3 : 7 (0.3)		6.01	80	205
	7 : 3 (0.7)		6.01	13	18
	3 : 7 (0.3)		19	250	328

**Table 2:** Parameters of the polynomial model representing the variation of the response (2-HBP concentration) in ED1 and ED2, each at 3 h and 6 h. *β*_0_, the experimental value at the center of the experimental domain; *β*_1_ and *β*_2_, parameters of factors 1 and 2 [ED1: *X*_1_ – the ratio of *n*-heptane: water, between 1 : 9 (0.1) and 9 : 1 (0.9), and *X*_2_ – initial DBT concentration in the organic phase, between 0.250 mM and 2 mM; ED2: *X*_1_ – the ratio of *n*-heptane : water, between 0.1 and 0.9, and *X*_2_ – cell (biocatalyst) concentration in the water phase, between 5 g L^−1^ and 20 g L^−1^ (DCW)]; *β*_1,2_, parameter of the interaction of the factors 1 and 2; *β*_1,1_ and *β*_2,2_, self-interaction parameters

Experimental design		ED1		ED2	
Response		2-HBP		2-HBP	
		3 h	6 h	3 h	6 h
Model parameters	*β*_0_	30.53	54.28	39.63	49.52
	*β*_1_	−217.57	−249.93	−225.39	−254.33
	*β*_2_	50.48	52.89	51.47	40.24
	*β*_1,2_	−84.09	−89.08	−93.63	−61.97
	*β*_1,1_	246.39	262.01	230.04	221.53
	*β*_2,2_	−30.67	−40.04	−8.02	55.3
Model validation (Fischer test)	Effectiveness of the parameters	18.87	21.36	26.25	516.37
	Significance level (*α*), *F* (5,8)	0.001	0.001	0.001	0.001
	Lack of fit	1859.68	3158.97	13.92	4.27
	Significance level (*α*), *F* (1,7)	0.001	0.001	0.05	>0.05
*R* ^2^	Coefficient of multiple determination	0.92	0.93	0.94	>0.99

In ED1, the highest result was obtained with 1 : 9 ratio of *n*-heptane : water (*i.e.*, 10% fuel) and 1.13 mM DBT, after 6 h (625 μM of 2-HBP) (Table 1), with complete DBT desulfurization. While in ED2, using a model fuel with a constant 1.13 mM DBT, the best result was also obtained with 1 : 9 ratio but with 12.5 g L^−1^ biocatalyst, after 3 h (534 μM of 2-HBP) (Table 1), also with complete DBT desulfurization. In both EDs responses were very similar. The most influential factor was always the *n*-heptane : water ratio, and its increase showed a negative influence in 2-HBP production, especially for ratios above 1 : 1. This can be seen in Table 1, where a ratio reduction from 1 : 1 to 1 : 9 always resulted in an increase of more than 10-fold in BDS. This is reinforced by the almost vertical lines in Fig. 2A–D, which are more prevalent when the ratio is below 1 : 1, and it is further made evident by the *β* values presented in Table 2, where *β*_1_ is always negative and at least 4 times larger (in absolute value) than *β*_2_. On the other hand, increasing concentrations of DBT or biocatalyst had a smaller but positive influence in the response for ED1 and ED2, respectively. For ED1 the increase of DBT from 0.37 mM to 1.88 mM, with 3 : 7 and 7 : 3 ratios, respectively, resulted in an increase of 2-HBP concentration of 4.8- and 2.6-times after 3 h, and 3.4- and 1.9-times after 6 h (Table 1). For ED2, the increase in biocatalyst concentration from 6 g L^−1^ to 19 g L^−1^ resulted in an increase of 2-HBP concentration of 3.1- and 1.6-times after 3 h, and 1.6- and 1.9-times after 6 h, with 3 : 7 and 7 : 3 ratios, respectively. Fig. 2A–D also reveals that BDS increases from the lower to the upper quadrants, for ratios below 1 : 1, and Table 2 confirms this observation, as *β*_2_ is always a positive number. The simultaneous increase of both factors, in either ED1 or ED2, has a negative influence in the response, though smaller when compared to the variation with the *n*-heptane : water ratio by itself, indicating a mitigation of the negative effect of greater *n*-heptane : water ratios by increasing either DBT or biocatalyst concentration. Finally, increasing reaction time had an overall positive effect, as BDS increased from 3 h to 6 h, despite most desulfurization occurring during the first 3 h. Three conditions were the exceptions to these observations: 1 : 9 ratio and 12.5 g L^−1^ cells in ED2 (where DBT was fully desulfurized after 3 h); 9 : 1 ratio and 1.13 mM DBT in ED1; and 3 : 7 ratio and 6.01 g L^−1^ cells in ED2 (where most desulfurization occurred from 3 h to 6 h).

Combining the results from both EDs, it becomes apparent that greater biocatalyst concentrations can be used to mitigate the negative effect of slightly higher fuel ratios (up to 30%) and that, contrary to what could be expected, an increase in DBT concentration, at least within the experimental domain (up to 1.88 mM), does not negatively influence BDS.

Furthermore, these results also indicate that the reduction in BDS with larger *n*-heptane : water ratios is most likely due to reduced bioavailability, and not necessarily related to toxicity caused by the organic solvent or DBT/2-HBP concentration, which is well below the IC_50_.^26^ Comparing the results obtained with ratios above 1 : 1 in both EDs (Table 1 and Fig. 2), it is possible to see that they are very similar for similar ratios, and that cells respond in a similar manner with the increase in either DBT or biocatalyst concentration. At higher ratios, there are not only lower BDS values, but also reduced influence of all factors. This could result from insufficient mixing within the tubes, which hinders contact between cells and DBT, thus lowering BDS regardless of time or cell concentration. At lower ratios, there is more water and less *n*-heptane, making it easier to form emulsions between both layers, thus explaining why for ratios ≥1 : 1 there is little influence of the factors and response is lower. By increasing either DBT or biocatalyst concentration for the same ratio, there is a greater chance of a DBT molecule reaching a cell. Furthermore, if a toxic effect was occurring some of the values in ED2 would be significantly greater, as more cells in the presence of the same amount of toxic substances would result in a higher number of metabolically active cells. This would be more obvious with longer reaction times, since more cells would be affected by the toxic substances, and thus the difference between both EDs should be greater, favoring ED2, while in fact at 6 h values are closer.

Considering exclusively the 2-HBP concentration, with this batch setup the best approach to optimize BDS up to 2 mM DBT, would be to use 10% fuel (0.1 or 1 : 9 ratio) and 90% cell suspension, at 20 g L^−1^, for 6 h. Nevertheless, in terms of future application, the utilization of 10% fuel is objectively inefficient, and it is likely to make the process unsustainable. To mitigate this issue different strategies could be applied to increase mixing efficiency, and allow for the use of greater fuel ratios without compromising BDS. In addition to increasing agitation speed, or biocatalyst concentration, some authors propose the use of immobilized biocatalysts, different bioreactor designs, or the incorporation of surfactants/emulsifiers into the process.^18,22,42–49^ The latter are probably amongst the most promising approaches to efficiently increase mass transfer, since they facilitate/prolong emulsion formation, increasing the area of contact between aqueous and organic phase, and facilitating the diffusion of hydrophobic compounds such as DBT, which consequently increases BDS activity of the biocatalysts.

**Fig. 3:**
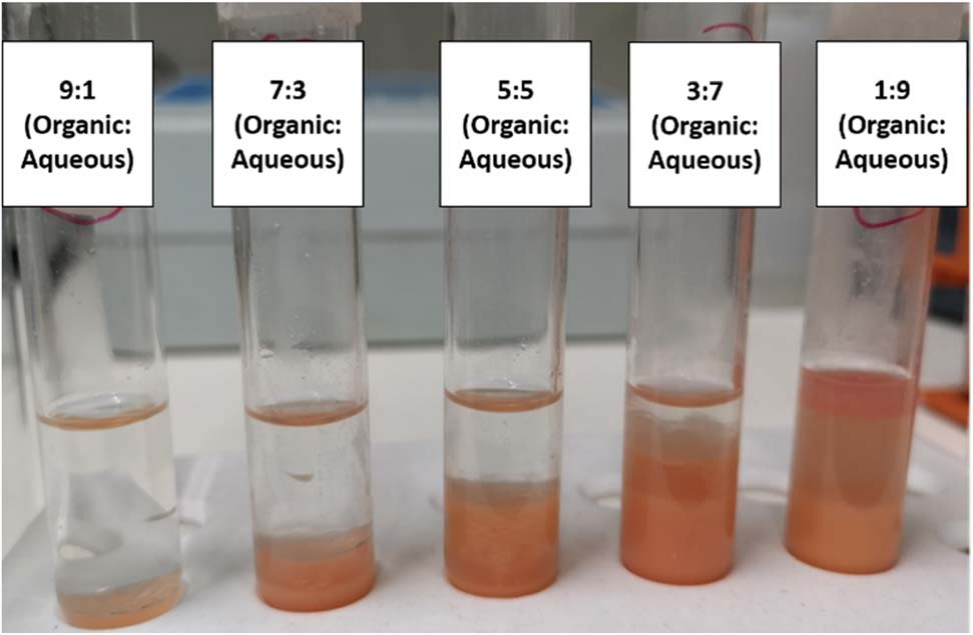
Mixtures of model fuel (*n*-heptane with DBT) with cell suspension (biocatalysts), at different organic : aqueous ratios, after 6 h of BDS. The photo was taken 1 h after shaking stopped to observe the phase separation/emulsion formation in each test condition.

However, as previously stated, BDS has a significant bottleneck in phase separation, which greatly hinders post processing. In this context, it is also important to look at BDS results considering emulsion formation. Thus, Fig. 3 intends to demonstrate the influence of organic : aqueous ratio on subsequent phase separation, after BDS process. This figure shows the different mixtures of model fuel (*n*-heptane with DBT) + water with cells (biocatalysts), tested in ED1, 1 h after shaking had stopped. It is clear that *n*-heptane : water ratios ≥3 : 7 (30% fuel) have much smaller emulsion, with clear demarcation of both phases, while 1 : 9 ratio (10% fuel) presents a strong emulsion, requiring intense centrifugation for recovery of the *n*-heptane, with substantial loss of the organic phase. These results reinforce what was already stated above, *i.e.*, that BDS activity, within the current experimental domain, seems to be more affected by the mixing efficiency than by any toxic effect of the model fuel (*n*-heptane with DBT), pointing to a better mixing at lower ratios.

Nonetheless, taking into consideration both BDS results and phase separation efficacy, 30% fuel (3 : 7 ratio) gains a greater interest, as an ideal ratio, because it ensures easier recovery of the organic phase. Moreover, comparing the amount of fuel treated and the differences in BDS (Table 1 and Fig. 2), its advantages are even more evident, since an increase of 3 times in fuel volume results in a reduction of 2-HBP production between 2.7 and 1.6 times, depending on the ED selected. Hence, this points to 30% fuel and 70% cell suspension (biocatalysts), at 20 g L^−1^, for 6 h, as the ideal conditions for BDS of at least 2 mM DBT in this batch setup.

Overall, these results demonstrate that *G. alkanivorans* strain 1B can act as a biocatalyst for BDS in biphasic systems and allowed the establishment of some parameters necessary to develop a continuous BDS system.

#### 3.2.1. Statistical validation

To evaluate the adequacy of the models to the sets of data, two tests were performed: (i) *F*-test for the effectiveness of the factors, to confirm if the source of variance included in the residuals, results from an inadequacy of the models to reproduce experimental data; and (ii) *F*-test for the lack of fit, to detect if the origin of the variance was a result of experimental error. The results are presented in Table 2 including the Fisher variation ratios and levels of confidence evaluated for each *F*-test. Significance levels below 0.05 were considered significant.

The *F*-test for the effectiveness of the factors indicates a level of confidence at which the null hypothesis (*H*_0_) can be rejected of at least 0.1%, for all ED's. Thus, it can be assumed with a good level of confidence that a significant amount of variance in the data has been represented by the factors in the models. This means that the factors, as they appear in the model, have an effect upon the responses analyzed. The F-ratio for the lack of fit indicates that for ED2 the source of variance contained in the residuals was explained by the experimental error at the significant level of 0.01 for 3 h and 0.05 for 6 h. For ED1, the null hypothesis can be rejected with a significance level greater than 0.001, showing that the lack of perfect prediction of the models cannot be explained by the experimental error. This is likely the result of a very low variance between replicates. Nonetheless, the model correctly represents general trends, especially for greater response values, as highlighted in the previous section, thus serving its purpose in the present study. To finish, model analysis through the coefficient of multiple determination (*R*^2^) was also performed (Table 2). The *R*^2^ values obtained show that only a limited amount of the sum of squares corrected for the mean is accounted for by the residuals (8% ED1-3 h, 7% ED1-6 h, 6% ED2-3 h and >1% ED2-6 h). When *R*^2^ approaches 1, there is a better fit between the empirical model and the actual data. The smaller *R*^2^ becomes, the less relevance the dependent variables in the model have in explaining behavior variation. All *R*^2^ values are ≥0.92, so it can be said they have a good fit.^50^

### 3.3. Characteristics of the continuous BDS system prototype

The innovative setup, already described in section 2.5 and presented in Fig. 1, is a bench-scale continuous BDS system, based on an integrative process coupling three sequential steps: (1) continuous biocatalyst production, (2) continuous biodesulfurization and (3) continuous separation. It was developed envisioning different objectives, namely: to streamline the BDS process, reduce biocatalyst production costs, increase BDS rates, and facilitate the downstream separation of cells, water and fuel.

There are few works describing continuous BDS setups, since most research is centered on small-scale batch assays. Amongst the continuous BDS systems employing free cells, most use the S-source present in the fuel directly for biocatalyst production, operating with continuous growing cell cultures.^51–53^ However, this can lead to several problems, which greatly limit its long-term application. The presence of the biphasic system during cultivation could result in mass transfer issues limiting nutrient dispersion, while the different compounds present in the fuels could have toxic effects resulting in slower/unstable growth rates. The fuel itself could also act as an inducer increasing biosurfactant production,^14^ redirecting carbon from biomass production, which would result in the formation of stronger and more stable emulsions, hindering the correct mixing of biocatalyst and fuel, and delaying the separation step.^53^ Additionally, direct cultivation in the sulfur-rich fuels leads to greater contamination risk with different microorganisms, as previously mentioned, which can reduce process efficiency and may even lead to a loss of fuel quality due to hydrocarbon degradation.^28^

To bypass these limitations, this novel system was based on the separation of biocatalyst production and fuel biodesulfurization processes. Therefore, *G. alkanivorans* strain 1B was continuously produced in a chemostat, under optimal conditions, to achieve a stable production of biocatalysts (cells) with similar BDS activity. Culture medium and cultivation conditions, such as agitation and aeration, can be adjusted to increase biomass production, and operational conditions can be more easily controlled to avoid contamination issues.

The continuously produced, metabolically active, biocatalysts are then mixed with a continuous stream of sulfur-rich fuel in another vessel where the biodesulfurization occurs under optimal conditions (Fig. 1, no. 3). Biocatalysts act as resting cells, and, as such, BDS becomes independent from cellular proliferation, eliminating the need for extra nutrient supplementation. Cells and fuel (intended to be desulfurized) are maintained in contact only while the biocatalysts maintain high BDS activity, reducing the influence of the potential toxicity, product inhibition or microbial contaminants present in the fuel. This reduced contact time also reduces biosurfactant production and limits the formation of strong emulsions. Under these conditions, and unlike other designs, such as the bubble column or the traditional sequential continuously stirred tank reactor, there is no need for pH control or active aeration, which reduces emulsion formation and decreases evaporation of volatile fuel compounds, as well as fuel oxidation.^18,46^ There is also no active production of gas, which is more likely to occur with growing cells or processes of reductive BDS, since the gas collecting bag did not have to be emptied throughout the assays, despite the system being airtight. Furthermore, the more efficient mixing, with a central propeller, results in increased mass transfer with less intensive agitation, leading to the formation of fewer clogs, especially when compared to the shake-flasks or closed tubes.

The mixture of water, cells and fuel is continuously collected and transferred to a separation column (Fig. 1, no. 5). In this column, the mixture is separated due to differences in density, maintaining the fuel mostly in the upper part and the biocatalysts (water + cells mix) mostly in the lower part. The fuel is continuously collected through a surface level tube, while the biocatalysts are drained through the bottom of the column. In the prototype presented, a leveling tube (Fig. 1, (g)) allows the regulation of the volume inside de column, as well as the ratio between organic and aqueous phase, which is maintained between 0.3 (3 : 7 ratio) and 0.5 (1 : 1 ratio) to stimulate phase separation, as demonstrated by the EDs (illustrated in Fig. 3). Due to the different densities of water and fuel, it is possible to adjust the column ratio, by varying the height of the leveling system relatively to the column. Placing the level at a higher position will increase the proportion of aqueous phase in the column, while placing the level at a lower position will increase the proportion of organic phase. The separation column was also maintained at a controlled temperature, in this case 30 °C, to reduce viscosity.

Overall, this separation process allowed the recovery of ≈90% of volume without centrifugation, with 8% more being recovered with centrifugations using only 424×*g* for 10 min. The final 2% can likely be recovered from the biomass, with harsher centrifugation conditions. This results in relatively easy separation of water + cells from the fuel, minimizing post-processing, allowing the system to operate with larger volumes, without significant centrifugation or filtration procedures. Unlike conventional systems dependent on growing cells, it allows easier continuous separation of desulfurized fuel, achieving this without the need for complex reactor designs dependent on laminar flow or any form of immobilization.^47^

Fuels with greater viscosity and different microorganisms or culture conditions may lead to greater emulsions, which will limit phase separation. Hence, there might be a need to adjust conditions in the separation column, such as: increased temperature to reduce viscosity, or changes in pH to break emulsion. Larger separation columns could also be used to increase the retention time and allow separation.

### 3.4. Optimization of continuous BDS conditions using the novel system

To optimize operation conditions, a series of continuous BDS assays were performed using *n*-heptane with DBT as a model fuel. Three key factors were studied, namely: (1) working volume, to simultaneously assess different retention/biodesulfurization times, as well as the importance of a headspace; (2) *n*-heptane : cells ratio and (3) initial DBT concentration, to better understand the range of application of the system. The results were evaluated in terms of BDS yield (*Y*_2-HBP_), in μmol of 2-HBP produced per gram of biocatalyst (μmol g^−1^); percentage of DBT converted into 2-HBP; and maximum specific BDS rate (*q*_2-HBP_) for the biodesulfurization vessel, in μmol of 2-HBP produced per g of biocatalyst per hour (μmol g^−1^ h^−1^).

Considering the results obtained in ED2, attempts were made at producing greater concentrations of biocatalysts in continuous conditions, however, increases in C-source concentration above 20 g L^−1^, resulted in carbon redirection towards biosurfactant production, resulting in lower biomass production yields, and excessive foaming and emulsions, limiting further increases with the current setup (data not shown). Hence, biocatalyst production was maintained as described in section 2.5 (*e.g.*, flow rate: 0.049 ± 0.001 L h^−1^; concentration (DCW): 8.25 ± 0.75 g L^−1^).

#### 3.4.1. Working volume

**Fig. 4:**
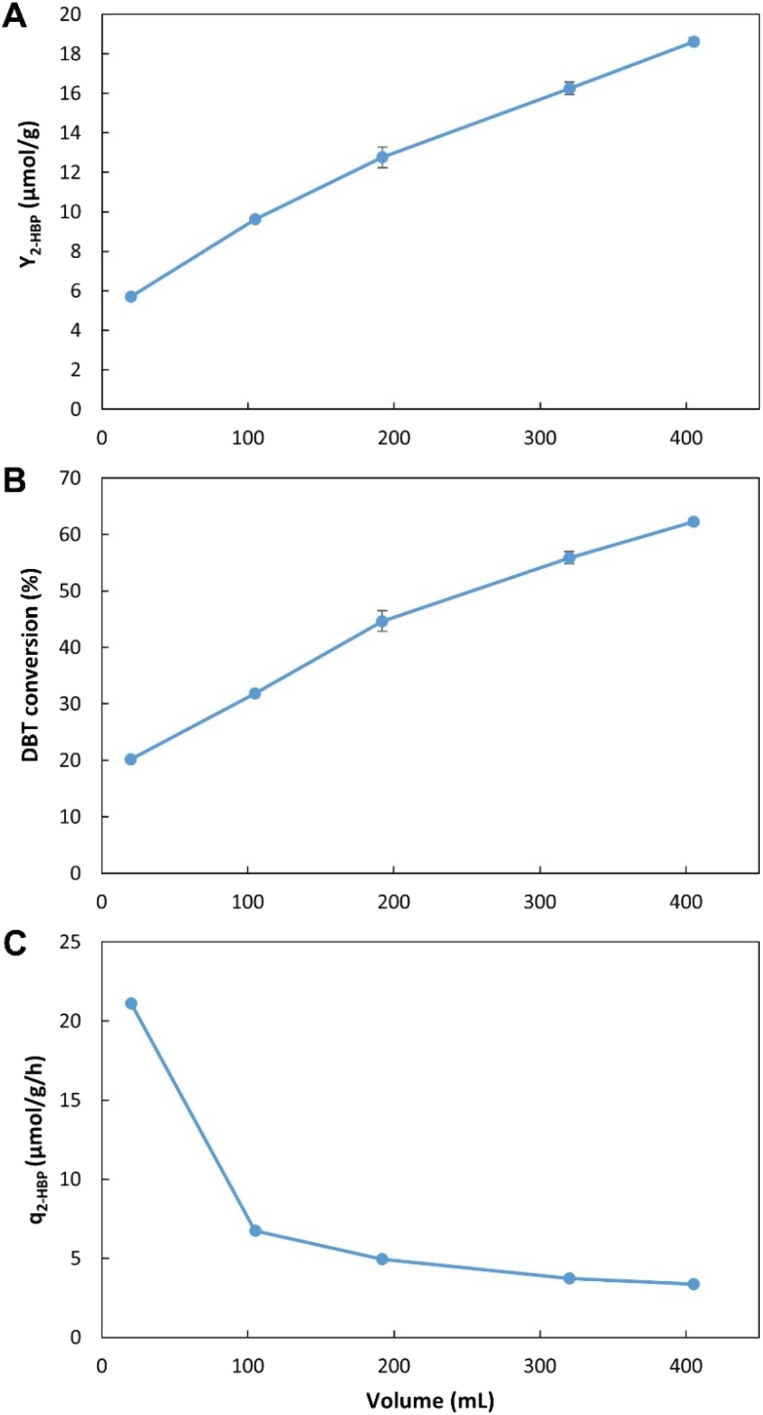
Influence of working volume on BDS using the novel continuous system, with 35% *n*-heptane with 500 μM of DBT (*i.e.*, 3.5 : 6.5 ratio). (A) – BDS yield (*Y*_2-HBP_), in μmol of 2-HBP produced per gram of biocatalysts (μmol g^−1^); (B) – percentage of DBT converted into 2-HBP; (C) – maximum specific BDS rate (*q*_2-HBP_) for the biodesulfurization vessel, in μmol of 2-HBP produced per gram of biocatalysts per hour (μmol g^−1^ h^−1^). Standard deviation (*n* = 2) is represented as error bars.

Fig. 4A–C presents the results obtained with the five different volumes tested (from 20 to 405 mL ± 6%), corresponding to five different retention times (from 16.2 min to 5.5 h). In terms of *Y*_2-HBP_, the results increased linearly with increasing volumes. The highest value obtained was 18.6 μmol g^−1^ with 405 mL and 5.5 h (Fig. 4A), corresponding to 62.3% of DBT conversion (Fig. 4B). In terms of *q*_2-HBP_, the results were reversed (Fig. 4C) with the highest recorded result being 21 μmol h^−1^ g^−1^ for the lowest volume (20 mL), corresponding to 16.2 min retention time, with values decreasing with increased retention time/volume.

These results allow for a direct comparison with the EDs results (Fig. 2, Tables 1 and 2). In the novel BDS system, within 5.5 h, using *n*-heptane with ≈0.5 mM of DBT, at 3.4 : 6.6 (0.34) ratio, the biocatalysts produced 333 μM of 2-HBP. This BDS value was greater than the results obtained in ED1. Despite having several advantages, such as a lower *n*-heptane : water ratio (3 : 7), greater DBT concentration (1.88 mM) and longer retention times (6 h), the batch assays (in ED1) presented lower BDS results (240 μM), as can be seen in Table 1, highlighting the advantage of the continuous system herein proposed.

These results also demonstrate that within the first 16.2 min the biocatalysts present the highest activity, however, they continue to maintain desulfurization abilities for longer than 5.5 h, resulting in increased *Y*_2-HBP_ despite the lower *q*_2-HBP_. There is a positive linear correlation between time and increase in *Y*_2-HBP_ (Fig. S2†). From a retention time of 16.2 min, up to 5.5 h, for each hour increased there was an increase of 2.4 μmol_2-HBP_ g_DCW_^−1^. This indicates that, at least up to 5.5 h, the system efficiency is positively influenced by greater retention times, thus confirming that there is little influence of toxic effects. Other than the initial BDS impetus, in which every cell started desulfurization simultaneously, further desulfurization rates are probably dependent on enzymatic reaction rates, and do not seem to be negatively affected by the retention time, at least within the interval tested. Furthermore, observing that the increase in volume corresponds to a decrease in headspace, and since there is no direct aeration, it is also possible to perceive that oxygen limitation does not seem to be responsible for any reduction in BDS activity, as it would lead to a reduction in *Y*_2-HBP_ for greater volumes/retention times.

These results could point to two different improvement strategies: (1) increase retention times to increase *Y*_2-HBP_, reducing the amount of biocatalyst needed; or (2) reduce retention times, to minimum values, to obtain the fastest desulfurization, but wasting biocatalyst with considerable BDS activity. Considering that biocatalyst production is broadly recognized as the most expensive step in the BDS process,^5,54^ the selection of optimal conditions must focus on obtaining the highest *Y*_2-HBP_, hence, it was determined that the maximum volume would be the optimum to proceed with the desulfurization studies. To operate with lower retention times, it would be necessary to include a step for biocatalyst recovery/recycling, which is not present in the current prototype, but can be explored in future works.

#### 3.4.2. Ratio of *n*-heptane : water

**Fig. 5:**
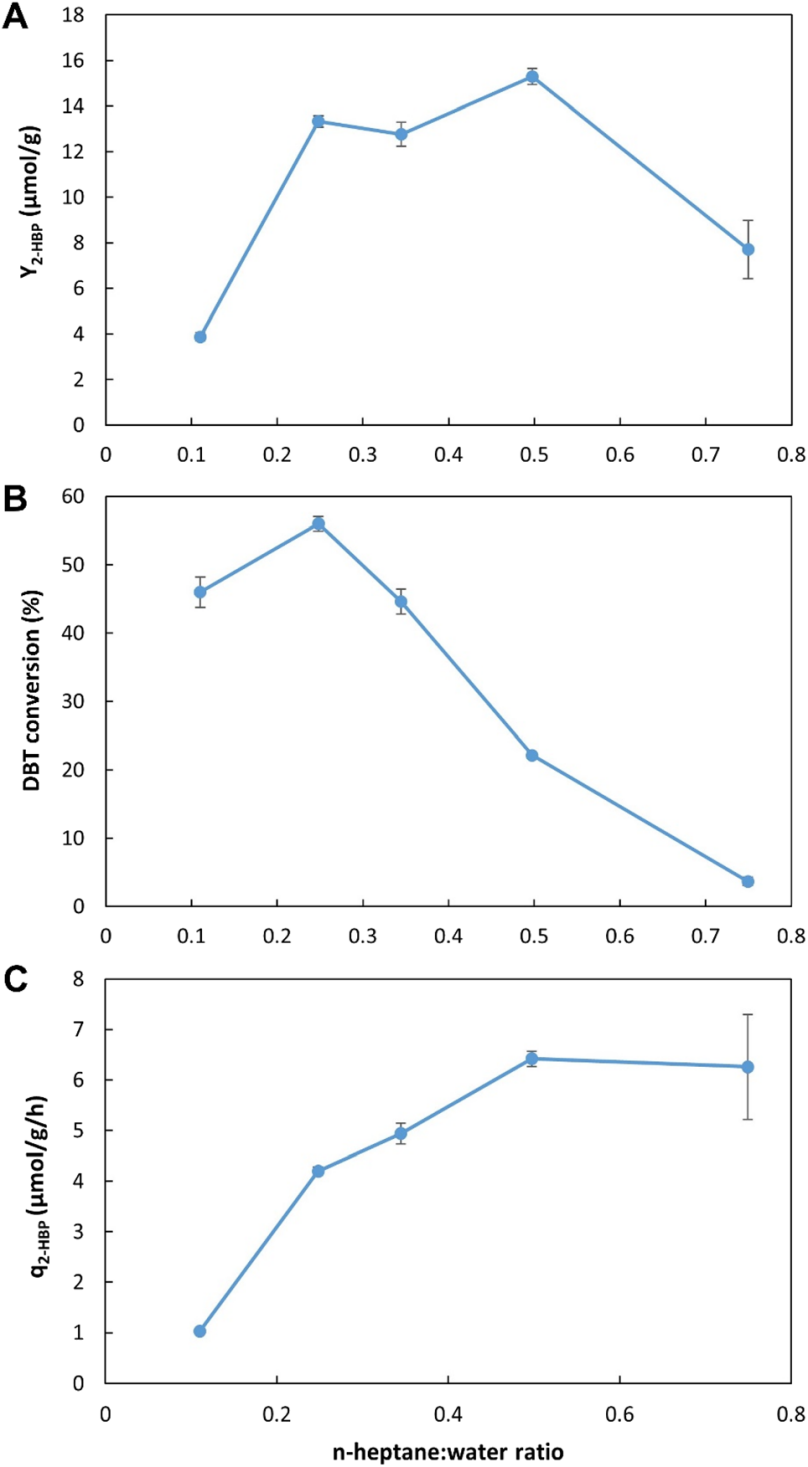
Influence of *n*-heptane (with 500 μM DBT) : water (with cells) ratio on BDS using the novel continuous system with a volume of ≈215 mL. (A) – BDS yield (*Y*_2-HBP_), in μmol of 2-HBP produced per gram of biocatalysts (μmol g^−1^); (B) – percentage of DBT converted into 2-HBP; (C) – maximum specific BDS rate (*q*_2-HBP_) for the biodesulfurization vessel, in μmol of 2-HBP produced per gram of biocatalysts per hour (μmol g^−1^ h^−1^). Standard deviation (*n* = 2) is represented as error bars.

Fig. 5A–C presents the results obtained when five different *n*-heptane : water ratios (from 1 : 9 (*i.e.*, 0.1 in *x*-axis) to 7.5 : 2.5 (*i.e.*, 0.75 in *x*-axis), with *n*-heptane containing 500 μM DBT and water containing the biocatalyst cells) were tested. In terms of *Y*_2-HBP_, the values were significantly lower for ratios below 2 : 8 (0.2) and above 1 : 1 (0.5). The highest value (15.3 μmol g^−1^) was observed for 1 : 1 (0.5) *n*-heptane : water ratio (Fig. 5A); however, it only represented 22% of DBT conversion (Fig. 5B), corresponding to 303 μM of 2-HBP production. The highest conversion was obtained between 1 : 9 (0.1) and 3.5 : 6.5 (0.35) ratios, with 60% conversion (116 μM of 2-HBP) at 2.5 : 7.5 (0.25) *n*-heptane : water ratio. The highest value of *q*_2-HBP_, was observed for *n*-heptane : water ratio ≥1 : 1 (0.5), with a value of 6.4 μmol g^−1^ h^−1^ (Fig. 5C).

These results reveal that, unlike what was observed for the closed tubes in batch assays, in the continuous desulfurization setup developed higher *n*-heptane : water ratios can be used without compromising desulfurization. Data from both EDs show that ratios of *n*-heptane greater than 1 : 9 resulted in lower concentrations of 2-HBP produced. In continuous BDS system assays, there is no direct comparison between these results and the ones obtained in the batch assay; however, looking at ED1 results (Table 1), when the ratio increased from 1 : 9 to 3 : 7, despite increasing DBT concentration (from 1.13 to 1.88 mM), BDS values were reduced to less than 50% (*e.g.*, from 625 to 240 μM, after 6 h). On the other hand, in the proposed setup, increasing the ratio from 1 : 9 to 2.5 : 7.5 led to a corresponding increase in 2-HBP concentration (from 235 to 303 μM). This seems to indicate that not only are the biocatalysts more active, since they are directly released into the biodesulfurization vessel without storage, but also that the mixing obtained with the central propeller could be more efficient at preventing the formation of clogs, which would hinder the interactions between biocatalysts and fuel.

Overall, and considering all values presented, it was determined that a value of 3.5 : 6.5 (0.35) *n*-heptane: water ratio would be ideal. It is within the optimum range for the highest *Y*_2-HBP_, without significantly compromising DBT conversion, while being above 3 : 7 (0.3) that is essential for better phase separation.

#### 3.4.3. Initial DBT concentration

**Fig. 6:**
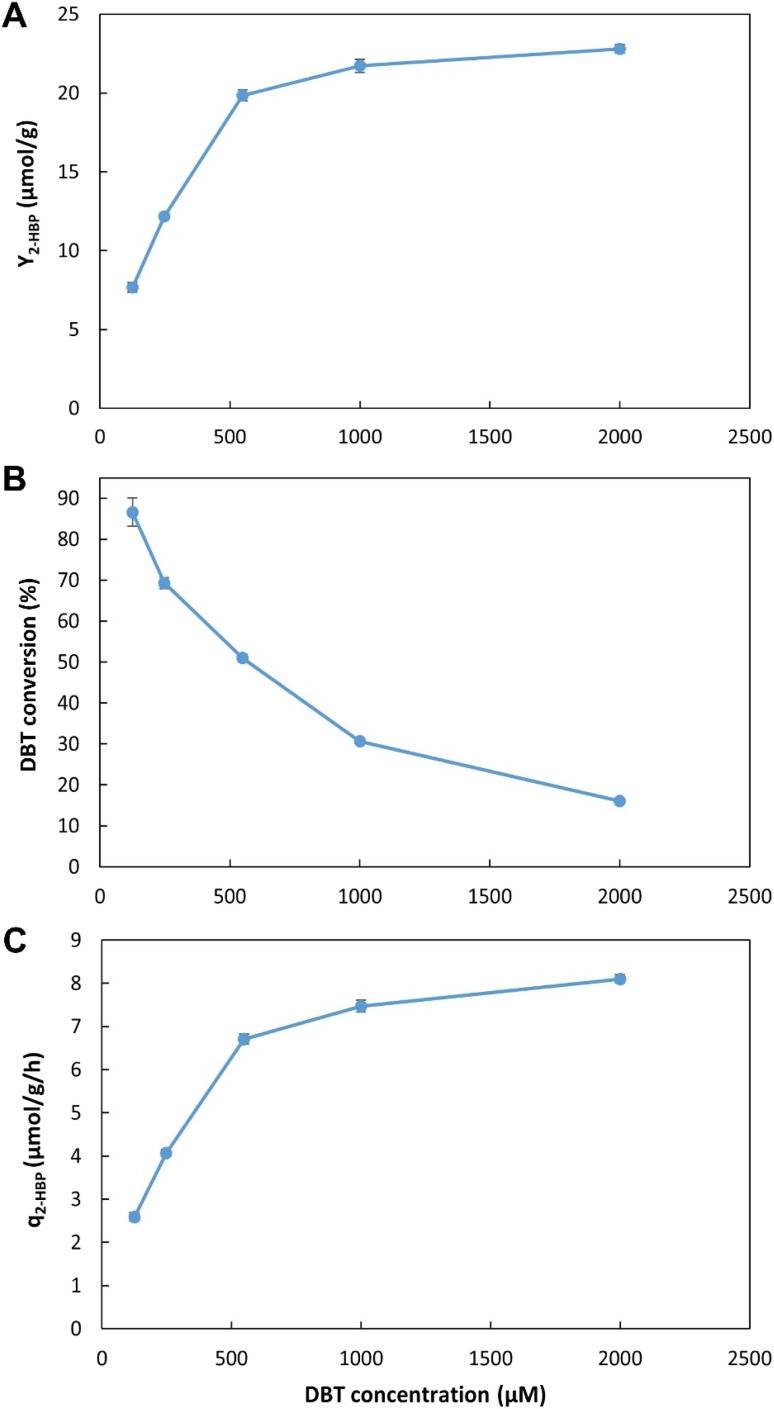
Influence of initial DBT concentration on BDS using the novel continuous system, with 35% *n*-heptane with 500 μM of DBT (*i.e.*, 3.5 : 6.5 ratio) and ≈215 mL of working volume. (A) – BDS yield (*Y*_2-HBP_), in μmol of 2-HBP produced per gram of biocatalysts (μmol g^−1^); (B) – percentage of DBT converted into 2-HBP; (C) – maximum specific BDS rate (*q*_2-HBP_) for the biodesulfurization vessel, in μmol of 2-HBP produced per gram of biocatalysts per hour (μmol g^−1^ h^−1^). Standard deviation (*n* = 2) is represented as error bars.

Fig. 6A–C presents the results obtained using five different initial DBT concentrations (from 125 μM to 2 mM). Both *Y*_2-HBP_ and *q*_2-HBP_ increased with the increase in DBT concentration (Fig. 6A and C, respectively). The greatest increase was observed in the range from 125 μM up to 500 μM, where *Y*_2-HBP_ increased from 7.7 to 19.9 μmol g^−1^ and *q*_2-HBP_ increased from 2.6 to 6.7 μmol g^−1^ h^−1^. Further increases in DBT concentration had a progressively lower effect, reaching a maximum *Y*_2-HBP_ of 22.8 μmol g^−1^ and *q*_2-HBP_ of 8.1 μmol g^−1^ h^−1^ with 2 mM of DBT, indicating that additional increases would have residual influence. In terms of total conversion percentage (Fig. 6B), the results were the opposite, attaining 86.6% conversion of DBT into 2-HBP with 125 μM of DBT, but only 16.1% conversion with 2 mM of DBT.

As observed for the working volume data set, these results also allow a direct comparison with the previous ED results. In the continuous BDS setup, using a similar biocatalyst concentration, for 2.8 h, with 3.5 : 6.5 *n*-heptane : water ratio at 2 mM of DBT, the maximum 2-HBP concentration obtained was 321 μM. In ED1, under similar conditions, even with more than twice the reaction time (6 h), the maximum 2-HBP concentration attained was only 240 μM. Thus, these results reinforce the notion that the biocatalysts present greater metabolic activity due to reduced down-times, since there is no need for storage, transport, and/or processing. Cells are immediately used as soon as they are produced with evident positive results. Furthermore, these results seem to indicate that using a biphasic system there is no inhibitory effect caused by DBT concentration within the range of the retention times and concentrations tested. The maximum value observed is probably linked to the concentration of biocatalysts (cells) and might be greater at higher concentrations. Additionally, higher DBT concentrations are not expected to have a negative influence on desulfurization, at least until values closer to IC_50_ are used (>44 mM, corresponding to more than 2000 ppm of sulfur).^26^

### 3.5. Sequential desulfurization

**Fig. 7:**
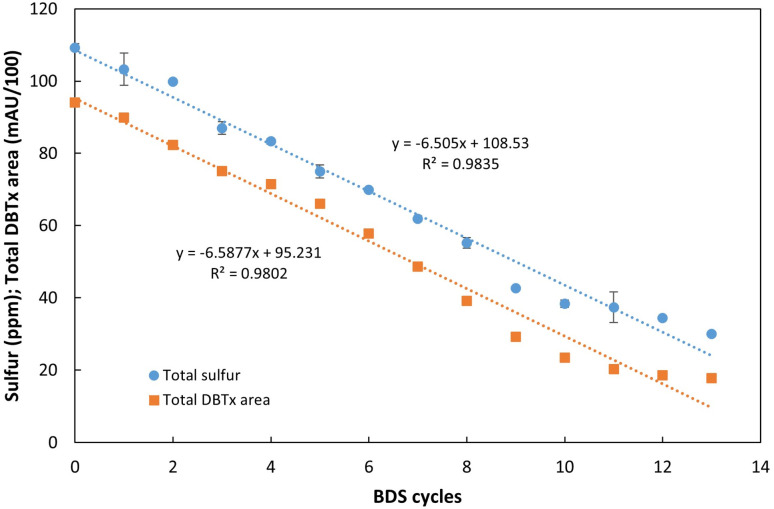
Influence of the number of desulfurization cycles for continuous BDS of an extremely recalcitrant model fuel, DBTx (mix based on *n*-heptane with equal parts of DBT, 4-mDBT, 4,6-dDBT and 4,6-deDBT), with 109 ppm of total sulfur. The results were evaluated in terms of total sulfur concentration (ppm) and combined area of the 4 DBTs mix (DBTx) (mAU/100). Standard deviation (*n* = 2) is represented as error bars.

**Fig. 8:**
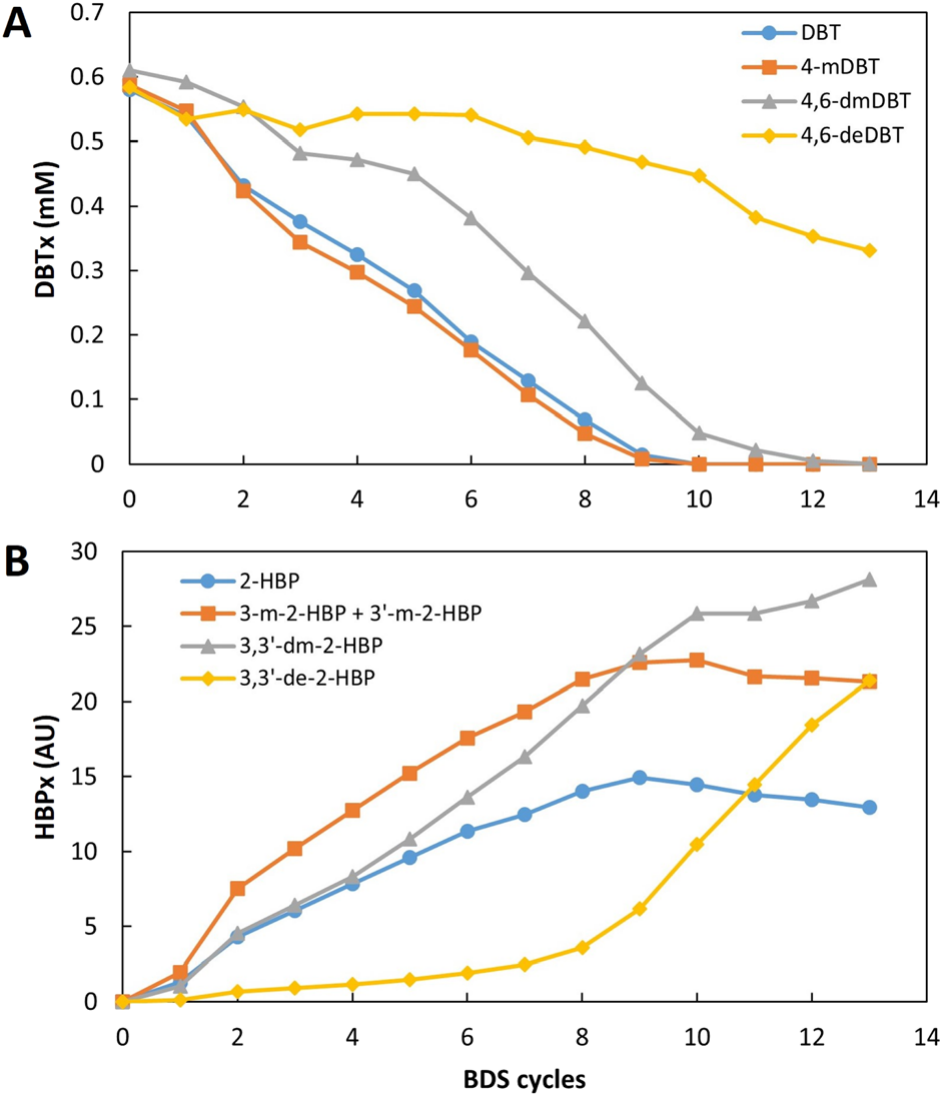
Influence of the number of desulfurization cycles for continuous BDS of an extremely recalcitrant model fuel, DBTx (mix based on *n*-heptane with equal parts of DBT, 4-mDBT, 4,6-dDBT and 4,6-deDBT), with 109 ppm of total sulfur. The results were evaluated in terms of: (A) individual DBTx components consumption (mM), and (B) individual peak area (AU) of the respective 2-HBP (HBPx) produced during BDS.

The results obtained revealed that it was possible to continuously convert a fixed amount of DBT into 2-HBP in a continuous setting. However, single step BDS was clearly below what is needed for commercial application, hence, a different approach was tested, by subjecting the same fuel to several BDS cycles, simulating a sequential process with multiple bioreactors. The assays were performed with a working volume of 405 ± 10 mL and a fuel ratio of 3.5 : 6.5 (35% fuel), as established in the previous section. An extremely recalcitrant model fuel was prepared using *n*-heptane with a mixture of DBTs (designated as DBTx): DBT, 4-mDBT, 4,6-dmDBT and 4,6-deDBT, each in equal parts, corresponding to approximately 109 ppm of total sulfur. This fuel was repeatedly fed to the continuous BDS system for a total of 13 cycles. At the end of the assay, samples from each cycle were analyzed in terms of total sulfur through XRF, and organosulfur compound profile through HPLC. Results are presented in Fig. 7, 8A and B.

As can be seen in Fig. 7, sulfur removal was successful and constant within these 13 BDS cycles, occurring at an average rate of 6.5 ppm per cycle. After 13 rounds, the sulfur content of the fuel decreased from 109 ppm to 30 ppm, corresponding to 72% reduction in less than 72 h of actual desulfurization, since the retention time was approximately 5.5 h.

Analyzing the results in terms of DBTx BDS (Fig. 8), it is possible to see that desulfurization of all organosulfur compounds started simultaneously (Fig. 8A), since the different HBPs (designated as HBPx), resulting from the desulfurization of DBTx, were detected after the first BDS cycle (Fig. 8B). However, the desulfurization of each compound showed different behaviors. DBT and 4-mDBT desulfurization was preferential, and occurred simultaneously at similar rates, with both DBT and 4-mDBT reaching full desulfurization after 10 cycles. 4,6-dmDBT started at a lower rate, and only in the 6th cycle, when DBT and 4-mDBT represented less than 50%, did it increase desulfurization rate, reaching more than 99.9% conversion by the 13th cycle. Finally, 4,6-deDBT maintained a residual desulfurization until the 7th cycle, as can be seen by the respective HBP (Fig. 8B), reaching 43% desulfurization by the 13th cycle.

Comparing total sulfur results with the sum of the 4 DBTs' peak areas (Fig. 7), it is possible to observe that they follow a similar trend, showing that the conversion of the organosulfur compounds is resulting in a corresponding desulfurization of the model fuel. However, it also becomes evident that there is a slight discrepancy between the expected S ppm value based on DBTx concentration and the measured value. The difference starts to increase at the 7th cycle and reaches its maximum at the 11th and 12th cycles, reducing by the 13th cycle. This behavior could result from the release of sulfur containing metabolites, however, given that there is no observable accumulation over time, these metabolites are most likely consumed in the subsequent cycles.

If all conditions were maintained constant, further cycles would likely continue to lower sulfur concentration, however, due to volume constrains, it was impossible to continue the assay while providing accurate results, as conditions were no longer comparable. It is important to note that this system was kept continuously working for more than 2 months to gather these results, and that it is dependent on the maintenance of the biocatalyst production reactor. Each cycle demands large volume sampling to ensure correct analysis, and a minimum volume is needed to fill the biodesulfurization vessel (Fig. 1, no. 3) and allow the continuous desulfurization to occur. In an industrial setting, several reactors can be sequentially assembled, with the fuel being transferred from one to another, reducing the operating time to the minimum necessary.

These results highlight the advantages of the new continuous system, with separation between biomass production and BDS. In prior batch assay results, there was a long lag-phase and BDS was only observed after 69 h.^31^ With the current set up, BDS occurred in every cycle despite the contact time being <6 h, indicating that there is no need for an adaptation period. Furthermore, there also seems to be a greater resistance to the toxic effects previously estimated, since HBPx concentration reached values above 0.62 mM, calculated as the value of the IC_50_ – 3 h for strain 1B growing cells.^26^ This means that, from the 5th cycle forward, even with the cells being exposed to concentrations that would inhibit more than 50% of the population within 3 h, no effect was observed on desulfurization. This probably results from the metabolic state of the cells being closer to that of resting cells, as conditions are not favorable for cell proliferation. These results also indicate that the continuous BDS approach may contribute to solve the problem of product inhibition. In traditional batch systems, the presence of 2-HBP inhibits BDS since it interferes with enzyme activity.^55^ The present work showed constant desulfurization activity through repeated cycles, giving no indication of product inhibition. It could be the case that, with these concentrations the short retention times do not favor enzyme inhibition. But further assays, at higher concentrations, are needed to confirm if this stays true for every condition.

In overall, these results show that *G. alkanivorans* strain 1B can act as a true biocatalyst since, within this range and as long as biocatalyst production was kept stable, desulfurization was constant, and mostly unaffected by the decrease in DBTx or the increase in HBPx. Moreover, these results are especially important because this model oil simulated an extremely recalcitrant fuel, where all S-sources present were difficult to treat through conventional methods. Most fuels would have a mixture of different sulfur compounds,^56,57^ many of which more easily metabolized than the ones used in this assay, with these recalcitrant forms being more prevalent in distillates, after concentration.

#### 3.5.1. GC-MS

In previous studies it was observed that strain 1B could convert DBT derivatives into other forms,^31^ however, the lack of analytical standards resulted in a putative identification based on the behavior of the BDS pathway with DBT and 2-HBP. Furthermore, it was also observed that 4-mDBT BDS resulted in the appearance of 2 different “HBP peaks”, which could indicate some deviation of the typical pathway. Hence, it was important to understand what the final BDS molecules were.^34^

**Fig. 9:**
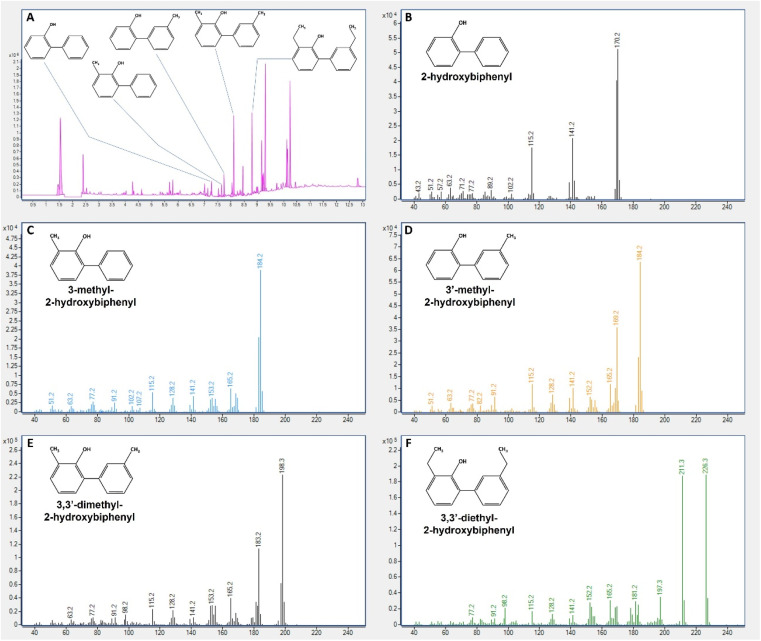
GC-MS analysis of the final biodesulfurized sample of DBTx model oil. (A) – GC chromatogram indicating retention time of each biodesulfurization product; (B) – 2-hydroxybiphenyl mass spectrum; (C) – 3-methyl-2-hydroxybiphenyl mass spectrum; (D) – 3′-methyl-2-hydroxybiphenyl mass spectrum; (E) − 3,3′-dimethyl-2-hydroxybiphenyl mass spectrum; (F) – 3,3′-diethyl-2-hydroxybiphenyl mass spectrum. Mass is in *m*/*z*, in *x*-axis of plots (B) to (F).

With this goal, the sample resulting from the 13th cycle was taken to a GC-MS to identify the compounds resulting from BDS. Fig. 9A–F illustrates the different results obtained, *i.e.*, the presence of the desulfurized forms from DBTx fuel. As expected, DBT was converted to 2-HBP, detected at 7.27 min (Fig. 9A), with a mass of 170 *m*/*z* (Fig. 9B); 4,6-dmDBT was converted to 3,3′-dm-2-HBP, detected at 8.11 min, with a mass of 198 *m*/*z* (Fig. 9E), corresponding to 2-HBP with two –CH_3_ groups; 4,6-deDBT was converted to 3,3′-de-2-HBP, detected at 8.803 min with a mass of 226 *m*/*z* (Fig. 9F), corresponding to 2-HBP with two –C_2_H_5_ groups. Lastly, 4-mDBT desulfurization resulted in the production of two peaks with different retention times (7.646 min and 7.742 min – Fig. 9A) but the same mass, 184 *m*/*z*, which corresponded to 2-HBP plus one methyl group. The analysis of the mass spectrum revealed the production of two different molecules 3-m-2-HBP and 3′-m-2-HBP. Contrary to the other three DBTs, 4-mDBT is an asymmetric molecule, and so, when the final step of desulfurization occurs, the –OH group can be placed either on the same ring as the methyl group or on the opposite ring, leading to two different end-molecules with the same mass. The mass spectrum shows that the peak detected at 7.646 min has a greater abundance of the 107 *m*/*z* mass which corresponds to a benzene ring with the addition of –CH_3_ and –OH groups (cresol minus one proton corresponding to the biphenyl), and 77 *m*/*z* mass corresponding to the benzene (minus the H from the connection) (Fig. 9C). This indicates that this peak corresponds to 3-m-2-HBP. The peak detected at 7.742, identified as 3′-m-2-HBP, was more abundant in the 169 *m*/*z* mass, corresponding to the loss of the –CH_3_ group (Fig. 9D). It could be the case that the presence of the –CH_3_ and –OH groups in the same ring offers some protection due to electrostatic interaction between groups and hinders the cleavage of these groups from the ring, promoting the cleavage of the two-ring connection. When both groups are separated, the methyl group is more exposed and easily cleaved, thus resulting in the appearance of the mass 169 *m*/*z*.

Furthermore, these differences were not exclusive to the retention time, since the two peaks presented different relative amounts in the GC analysis, and consistently presented different areas as detected by HPLC (data not shown). This indicates that the position in which the 4-mDBT molecules contact the BDS enzymes influences reaction speed. In fact, the production of 3′-m-2-HBP was always greater than the production of 3-m-2-HBP (>1.5 times). This is especially evident when 4-mDBT concentrations were greater (>3 times) and gradually decreases with the several cycles. Comparing both peaks, 3′-m-2-HBP has a much higher relative production in the first cycles when there is more 4-mDBT, but constantly reduces its production afterwards. On the other hand, 3-m-2-HBP presents a stable production over the different cycles, only reducing by the 9th cycle, when 4-mDBT is almost residual (suggesting that the reduction in production is caused by a reduction of availability). This indicates that there is no preference when accessing the molecule, but only a difference in BDS speeds. The enzyme can access the molecule at similar speed regardless of its relative position, but takes more time to process depending on the relative position of the methyl group. This explains why, regardless of 4-mDBT concentration (within minimal values), the production of 3′-m-2-HBP is kept stable throughout the cycles. Since time and biocatalyst concentration are always constant, desulfurization is only limited by reaction speed. This could also help to explain the extended lag-phase observed by Silva *et al.*,^31^ when cultivating the strain 1B with 4-mDBT as the sulfur source, or with the mixture of the four DBTs, which is not observed in the present results. In growing cells assays, strain 1B relies on 4-mDBT as S-source for growth, and, if 50% of the population accessed the 4-mDBT molecule in the slower relative position, this would result in a much lower BDS/growth speed, significantly hindering the whole process until either the 4-mDBT was fully transformed, or enough biomass exists to compensate this inhibition. This further highlights the importance of the continuous system, herein developed, as a BDS tool for handling complex multiple molecule sulfur mixtures (*e.g.*, pyrolysis/crude oils).

These results seem to be opposite to those presented by Chen *et al.*,^58^ which reported that 3′-m-2-HBP was the most abundant form resulting from the 4-mDBT BDS by *Gordonia* sp. SC-10. This fact indicates that the two bacteria have different behaviors regarding the 4-mDBT BDS, which had already been reported by Onaka *et al.*,^59^ in their study on desulfurization of asymmetric molecules, confirming that it could be a characteristic inherent to each desulfurizing strain.

### 3.6. BDS of real fuel mixtures

**Fig. 10:**
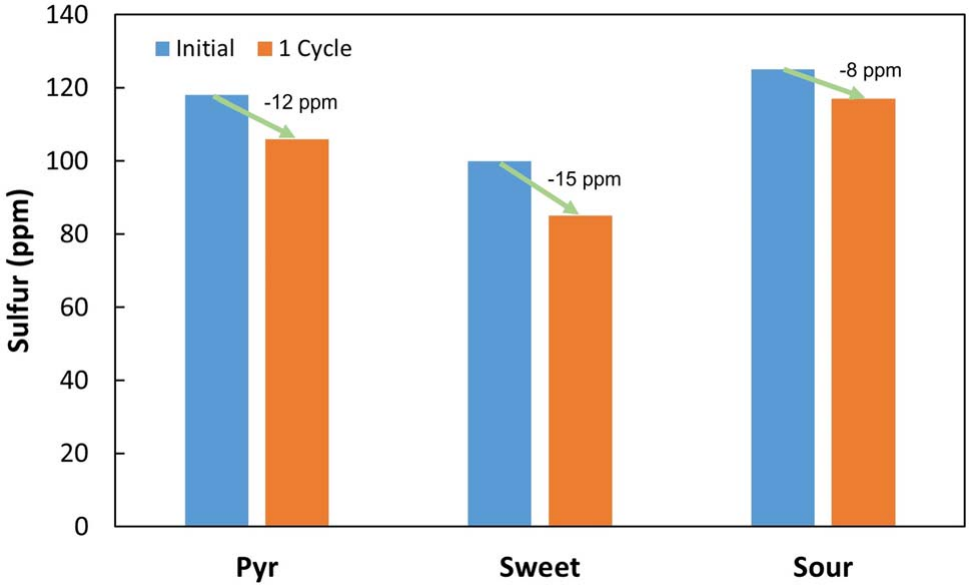
Total sulfur concentration (ppm) before and after one cycle of biodesulfurization on different real fuels, diluted in *n*-heptane to at least 100 ppm of initial sulfur. Pyr – pyrolysis oil; sweet – sweet crude oil; sour – sour crude oil.

To confirm system viability with actual fuels, three different assays were performed using real fuels: a tire/plastic blend pyrolysis oil (Pyr), a sweet crude oil (sweet), and a sour crude oil (sour). Due to volume constraints, the fuels were first diluted in *n*-heptane, to at least 100 ppm of initial sulfur and a single desulfurization cycle was tested. The results obtained are shown in Fig. 10.

All the three fuels presented higher desulfurization results than those observed for the DBTx mixture. Sour crude oil presented the lowest value, with a decrease of 8 ppm, while the pyrolysis oil and the sweet crude oil, presented a 12 and 15 ppm reduction in sulfur, respectively. As previously explained, it is uncommon for the sulfur content of a fuel to be entirely composed of DBT and other organosulfur compounds. Indeed, in unprocessed fuels, DBT and its derivatives represent only a fraction of the total sulfur. Therefore, this could justify the greater desulfurization rates when compared to those of the sequential assays for the model fuel tested (Fig. 7). In these real fuels, some of the sulfur compounds present will be simpler molecules, which are easier to transform, thus resulting in greater sulfur removal.

The differences between fuels could be explained in a similar manner, since Iranian light sour oil is known to be richer in more complex molecules,^60^ being more difficult to treat by conventional methods. Tire pyrolysis oils are also known to be abundant in DBT and organosulfur molecules,^61^ but since scrap tires only represented 30% of the original material blend, most of the sulfur compounds present could be simpler molecules originated from the plastic, and therefore easier to treat. Sweet crude oil is typically the simplest to treat, containing fewer complex organosulfur molecules,^62^ which explains the greater reduction within a single cycle. Lastly, it is important to point out that BDS values for each of the three fuels result from a single cycle with only 5.5 h of contact, due to the small amount of sample available. With more volume of each fuel available it would be possible to test several rounds and increase total desulfurization. Other works have reached greater BDS percentages in a single step, however, this process has the advantage of easier implementation and long-term application.

Furthermore, to the best of our knowledge, this is the first reported work that applies BDS to pyrolysis oil, in this case from a tire/plastic residues blend. With the increasing application of this thermochemical conversion technology, it is necessary to explore new approaches to make post-processing cleaner and cheaper. While BDS of heavy crude oils could be limited due to high concentrations of sulfur (typically several thousand ppm),^57^ BDS of pyrolysis oils might be easier to implement, since these oils have lower concentrations of sulfur, typically no more than a few thousand to several hundred ppm.^63^ Nonetheless, pyrolysis oils can be abundant in complex organosulfur molecules, therefore, before being commercialized, they will have to undergo desulfurization. So, since the presence of complex organosulfur compounds hinders HDS, BDS can be an interesting alternative.

## 4. Conclusion

This work presents the setup for the first continuous BDS system consisting of an integrated process, coupling continuous biocatalyst production, continuous BDS and continuous separation. Through it, cells of strain 1B were capable of desulfurizing both recalcitrant model fuels and real fuels, acting as true biological catalysts, maintaining a stable response over time, despite the presence of multiple complex organosulfur compounds and BDS products. Furthermore, by employing this novel system it was possible to mitigate/surpass several BDS limitations: (i) there was neither visible toxic effect from fuel nor inhibition by the final product (*i.e.*, increase in HBPx); (ii) there was an increase in mixing efficiency, which allowed the utilization of greater fuel ratios; and (iii) there was a reduction in problems associated with the separation step, with direct recovery of >90% of fuel without centrifugation. Additionally, it was the first time that the BDS process was used for pyrolysis oils, in this case from plastic/tire recycling. This reinforces the applicability of this system for real fuels BDS, and with further optimization of conditions, jointly with the assembly of several sequential reactors, it could be possible to establish a system that would continuously produce ultra-low sulfur fuels. This innovative BDS system is the first example of its kind and can be used as a tool to study and optimize biodesulfurization conditions in a more realistic approach, when compared to a final large-scale installation. BDS might become especially significant towards the production of new-generation fuels, such as pyrolysis oils, biodiesel, syngas or biogas. By integrating it in a biorefinery and taking advantage of the coproduction of high-added value products, it could be possible to make large-scale BDS a cost-effective viable process, allowing the exploration of sulfur-rich biomasses/residues, without compromising desulfurization costs, thus increasing the range of available options for energy production.

## Author contributions

T. P. S.: conceptualization, investigation, methodology, visualization, formal analysis, validation and writing – original draft preparation. S. M. P.: conceptualization, investigation, methodology, formal analysis, validation, supervision, writing – reviewing and editing, project administration and funding acquisition. J. T.: investigation (microbial bioreactor monitoring/maintenance). F. P.: investigation (GC-MS data analysis). T. C.: investigation (sulfur determinations in pyrolysis/crude oils in *n*-heptane matrix). J. C. R.: investigation (EDs data analysis). L. A.: conceptualization, investigation, methodology, formal analysis, validation, supervision, writing – reviewing and editing, project administration and funding acquisition.

## Conflicts of interest

The authors declare no competing interests.

## Supplementary Material

RA-014-D3RA07405F-s001
