# Supplementary material for: Streamlining the biodesulfurization process: development of an integrated continuous system prototype using Gordonia alkanivorans strain 1B
Source: RSC Adv. 2024 Jan 2;14(1):725–42. doi: 10.1039/d3ra07405f (PMC10758933; doi:10.1039/d3ra07405f)
Supplement: RA-014-D3RA07405F-s001 [file RA-014-D3RA07405F-s001.pdf]

Electronic Supplementary Material (ESI) for RSC Advances.
This journal is © The Royal Society of Chemistry 2023

SUPPLEMENTARY DATA

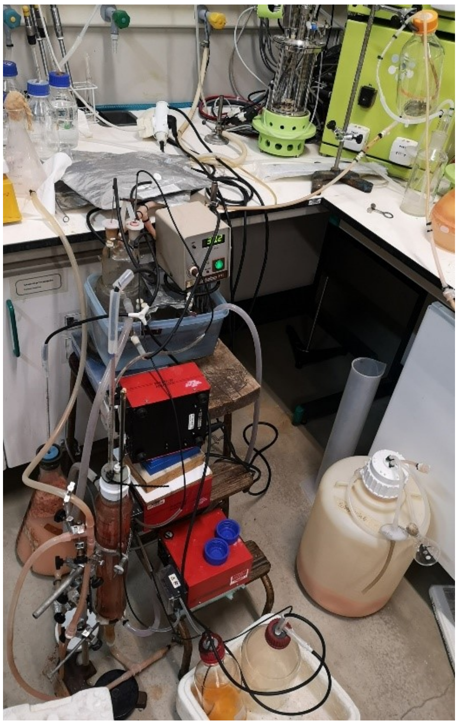

Fig. S1. Photo of the continuous biodesulfurization system prototype focusing on steps II and III.

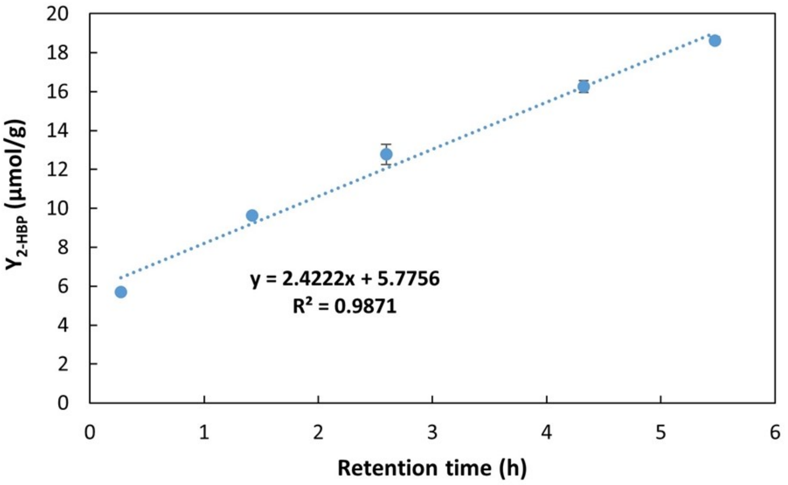

Fig. S2. Correlation between $Y_{2\text{-HBP}}$ and retention time, when different volumes were tested on the BDS continuous system, with 35% n-heptane containing 500 µM of DBT (3.5:6.5 ratio).
